# Genome-wide scan for common variants associated with intramuscular fat and moisture content in rainbow trout

**DOI:** 10.1186/s12864-020-06932-0

**Published:** 2020-07-31

**Authors:** Ali Ali, Rafet Al-Tobasei, Daniela Lourenco, Tim Leeds, Brett Kenney, Mohamed Salem

**Affiliations:** 1 Department of Animal and Avian Sciences, University of Maryland, College Park, MD 20742 USA; 2 Computational Science Program, Middle Tennessee State University, Murfreesboro, TN 37132 USA; 3 Department of Animal and Dairy Science, University of Georgia, Athens, GA 30602 USA; 4 National Center for Cool and Cold Water Aquaculture, Agricultural Research Service, United States Department of Agriculture, Kearneysville, WV USA; 5 Division of Animal and Nutritional Sciences, West Virginia University, Morgantown, WV 26506 USA

**Keywords:** Fat content, Moisture, GWAS, Single-step, QTL

## Abstract

**Background:**

Genetic improvement of fillet quality attributes is a priority of the aquaculture industry. Muscle composition impacts quality attributes such as flavor, appearance, texture, and juiciness. Fat and moisture make up about ~ 80% of the tissue weight. The genetic architecture underlying the fat and moisture content of the muscle is still to be fully explored in fish. A 50 K gene transcribed SNP chip was used for genotyping 789 fish with available phenotypic data for fat and moisture content. Genotyped fish were obtained from two consecutive generations produced in the National Center for Cool and Cold Water Aquaculture (NCCCWA) growth-selective breeding program. Estimates of SNP effects from weighted single-step GBLUP (WssGBLUP) were used to perform genome-wide association (GWA) analysis to identify quantitative trait loci (QTL) associated with the studied traits.

**Results:**

Using genomic sliding windows of 50 adjacent SNPs, 137 and 178 SNPs were identified as associated with fat and moisture content, respectively. Chromosomes 19 and 29 harbored the highest number of SNPs explaining at least 2% of the genetic variation in fat and moisture content. A total of 61 common SNPs on chromosomes 19 and 29 affected the aforementioned traits; this association suggests common mechanisms underlying intramuscular fat and moisture content. Additionally, based on single-marker GWA analyses, 8 and 24 SNPs were identified in association with fat and moisture content, respectively.

**Conclusion:**

SNP-harboring genes were primarily involved in lipid metabolism, cytoskeleton remodeling, and protein turnover. This work provides putative SNP markers that could be prioritized and used for genomic selection in breeding programs.

## Background

Fish are excellent source of protein with lower content of total fat, saturated fat, and cholesterol and higher omega-3 fatty acids compared to other animals. These characteristics make fish fillets an ideal source of nutrition according to a consensus dietary studies and recommendation [1]. Thus, fillet quality traits have economic importance to the aquaculture industry [2], and consumer attitude towards fish is influenced by fillet quality attributes [3]. For profitable aquaculture production, there is a need for fish fillets with optimum nutritional values and consistent organoleptic qualities. Rainbow trout fish fillet contains ~ 4–18% by weight fat. Rainbow trout cultured at Clear Springs Foods Inc. (Buhl, ID, USA), the largest producer in the U.S., contains fat content of 12–13% [4]. Variations in fat content can result in positive and negative impacts on fillet quality [4, 5]. Both the quantity and quality of intramuscular lipid impact fillet juiciness, flavor, color, texture, and shelf-life [5–8]. Selection on fat content can enhance fillet color and texture [9], feed conversion ratio (FCR), and protein-retention efficiency [10]. However, accumulating excessive lipids in the muscle makes fillet processing difficult and reduces fillet firmness [11–13]. In addition, high levels of polyunsaturated fatty acids make the fillet more prone to lipid oxidation, which contributes to the development of rancid flavor and changes in color and nutritional value [14]. Therefore, management of fat content in fish could be used to minimize the variation in eating quality and yield a product of predictable quality [6]. The aquaculture industry usually controls the fat content of fillets by adjusting lipid content in the diet [15–17]. However, there are limitations in using the dietary lipids approach without deteriorating fillet quality due to lipid oxidation and diminishing profitability due to increased feed cost and accumulation of fat in the viscera instead of the muscle. Also, a widely adopted culturing triploid in rainbow trout can prevents loss of fillet quality associated with fat mobilization and protein catabolism during sexual maturation [9, 18].

Fish fillet is a highly perishable food, at least partially, due to high moisture content (60–70%), which results in off-flavors and faster flesh spoilage because water facilitates enzymatic activity and bacterial growth [19, 20]. Low-temperature storage is used to control water activity. However, slow enzymatic reactions can still support microbial growth at low temperatures [21]. Previous studies showed a high correlation between fat and moisture content [22]. In mammals, the intramuscular fat content exhibits a significant negative correlation with moisture content [23, 24]. In fish, this correlation depends greatly on the energetic demands associated with various physiological conditions [18, 25, 26]. An antagonistic biological relationship between traits may hinder their simultaneous improvement, which could lead to unwanted changes in fillet quality [27, 28]. Therefore, an optimal balance among important economic traits needs to be established to enhance product quality and industry returns [2]. Knowledge of the heritability and genetic architecture of each trait provides information necessary in developing appropriate multi-trait selection programs.

Selective breeding can be used to enhance phenotypic traits of interest. A two-way program of selection on muscle fat content was initiated in rainbow trout to produce lean and fat lines where the fat percentage increased by ~ 15 to 31% in the fat line depending on the diet [9, 29]. These lines were used as a model to study the effect of muscle fat content on fillet quality [9]. Separately, five generations of family-based selection on body weight of rainbow trout were performed at the USDA NCCCWA [30]. In the third-generation (year class (YC) 2010), fish fillet fat content showed a moderate correlation with whole body weight (coefficient of determination R^2^ value of 0.50) [31]. Therefore, selection for bodyweight yielded heavier fish with more fat in the muscle. Similarly, gilthead seabream exhibited a 0.1% increase in muscular fat content concomitant with a 0.08% decline in moisture content per increment of ten grams in weight [32]. Muscle fat and moisture content showed moderate heritability in fish, including rainbow trout, implying the existence of genetic variance in a rainbow trout population selected for an enhanced rate of growth [33], thus making genetic responses to selection possible. However, in salmonids, the genetic architecture of fat and moisture content has not been fully explored in a genome-wide scale [34]. Understanding the genetic basis of the phenotypic traits in question and development of fish strains of improved genetic gain will enhance the efficiency of breeding programs, aquaculture industry profitability, and consumer satisfaction.

Genome-wide association (GWA) studies can identify large-effect variants responsible for phenotypic variations, which can be prioritized in genomic selection. A few GWA studies have been conducted on aquaculture species to identify quantitative genomic loci (QTL) responsible for the genetic variability in body weight [35], fillet quality [35, 36], and disease resistance [37]. In fish, a few GWA studies were performed on Atlantic salmon [11, 34] and common carp [38] to identify QTL associated with muscle fat content. In Atlantic salmon, few significant SNPs associated with muscle fat content were identified using a ~ 5 K and 57 K SNP panels [11, 34]. In common carp, a high-density, 250 K SNP array revealed eight SNPs related to muscle fat content; however, none of the SNPs surpassed the genome-wide significance level [38]. The two studies did not identify QTL explaining a large proportion of the genetic variance in fat content in fish. To the best of our knowledge, no GWA studies have been performed in rainbow trout to identify SNP markers associated with genetic variance for fat and moisture content.

A 50 K transcribed SNP-chip, suitable for GWA analyses, has been recently developed in our laboratory. The array has been used to identify large-effect QTL responsible for genetic variance in fillet yield, firmness, protein content, and body weight gain using the same fish population used in this study [36, 39, 40]. The current study aimed to identify QTL associated with the additive genetic variance in fillet fat and moisture content for the same rainbow trout population.

## Results and discussion

Muscle fat and moisture contents are interrelated attributes that affect the organoleptic quality and nutritional value of muscle foods [8, 38, 41, 42]. In fish, high-fat content may influence fillet processing and reduce the firmness leading to fillet downgrading [11]; moreover, it significantly impacts texture, juiciness, and flavor [5–7]. In mammals, increased marbling scores are positively related to beef tenderness, accounting for ~ 9% of the shear force variation [43]. The inability to retain moisture during postmortem storage, in both fish and mammals, is associated with a high drip loss and, in turn, reduces the industry profitability by influencing processing yield and palatability [44, 45]. In the pork industry, drip loss results in up to 10% product losses affecting profitability at wholesale and retail levels [44]. Similarly, 1.5 to 5% of drip losses were reported in salmon [46, 47]. Muscle quality traits in rainbow trout are complex and controlled by many genes (i.e., polygenic in nature) [31, 39]. Increased knowledge of the genetic basis of muscle quality traits will facilitate to advance the commercial breeding in salmonids. GWA studies are powerful tools to identify genetic variants associated with complex traits [36, 39, 40]. However, no GWA studies were previously conducted to dissect the genetic architecture of fillet fat and moisture contents in rainbow trout. The SNP-based heritability for fat and moisture content was 0.39 and 0.51, respectively, suggesting existence of adequate genetic variability in the NCCCWA fish population to allow genetic improvement through selective breeding. A higher rate of genetic gain is obtained when genomic information is used [48].

In this study, we used genomic windows of 50 SNPs of a 50 K SNP chip to perform GWA analyses, in addition to the single-marker analysis approach, to identify genomic regions associated with the traits. Given that the 50 K, SNP chip contains SNPs of potential association with intramuscular fat content, all fish used to build the SNP-chip were excluded from the GWA analysis in the present study.

**Fig. 1:**
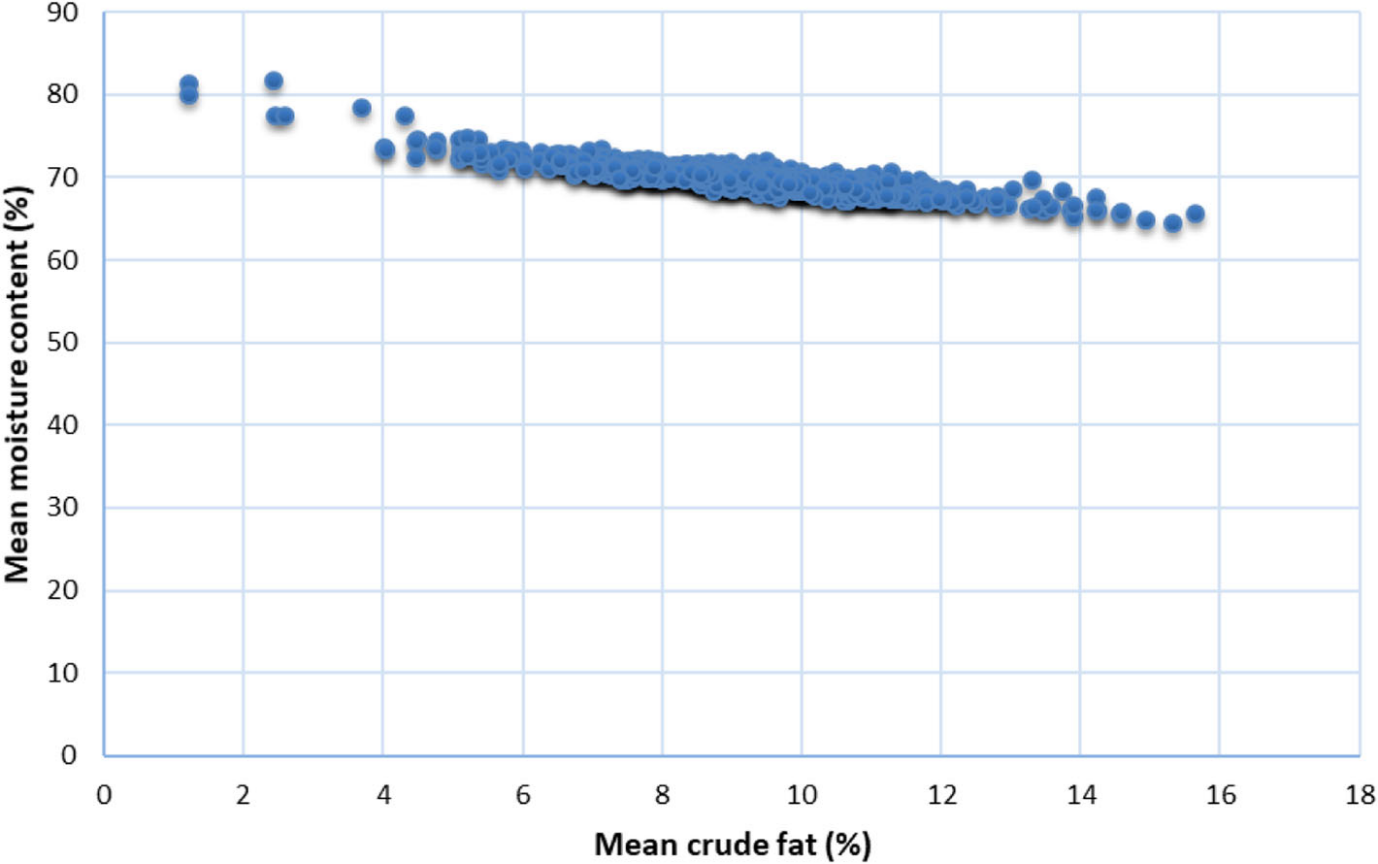
Reverse relationship between intramuscular fat and moisture content in fish used for GWA analyses

The fish population had an average muscle fat content of 9.2 ± 1.91 (%) and moisture content of 69.93 ± 1.75 (%). Variations in fat and moisture content are shown in Fig. 1. Previous studies reported a significant correlation between changes in fat and moisture content in fish [26, 32]. Consistently, our data showed a significant negative correlation between fat and moisture content (R = − 0.88; *p*-value = 6.3E-262). Further, fat content was regressed on moisture content using phenotypic data from separate year classes to make sure the size and age differences between fish from YC 2010 and YC 2012 are not influencing the correlation between the traits. Interestingly, the negative correlation between fat and moisture content (R = − 0.88) was maintained in fish from the two consecutive generations.

### QTL affecting muscle fat and moisture content using WssGBLUP

**Fig. 2:**
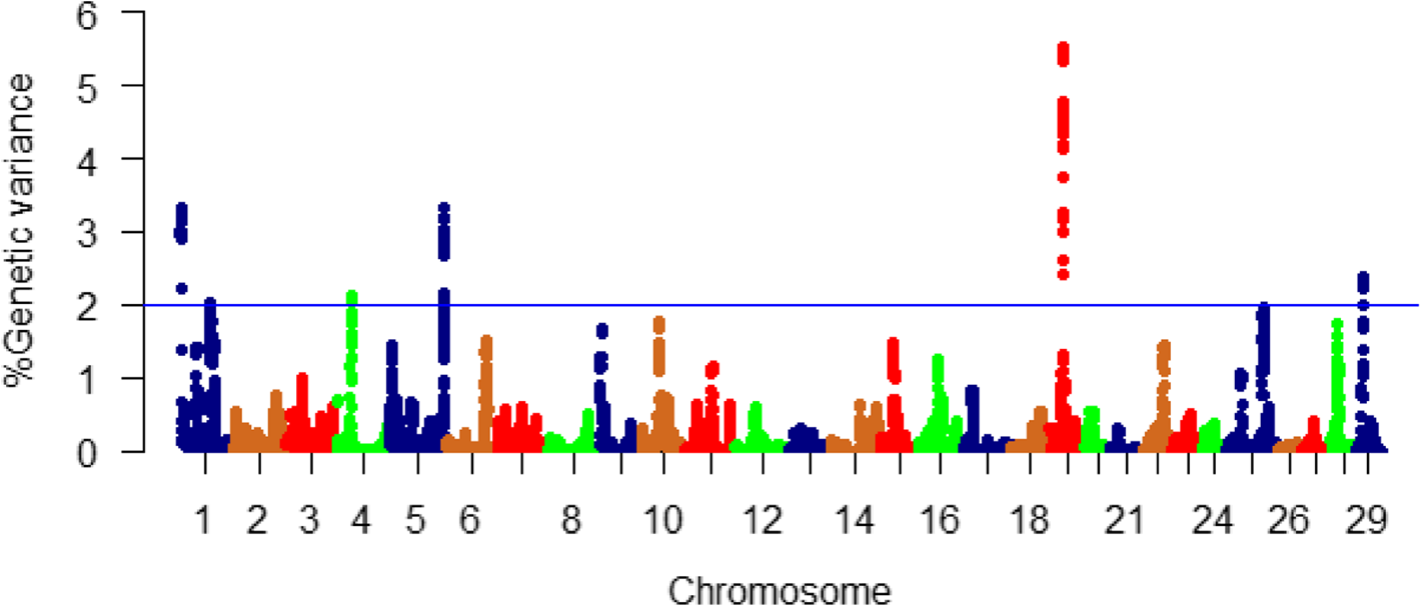
Manhattan plot showing association between 50 SNP-genomic sliding windows and muscle fat content. Chromosome 19 showed the highest peaks with genomic loci explaining up to 5.51% of the additive genetic variance. The basal blue line represents 2% of the genetic variance explained by the sliding windows

All 35,322 SNPs (70.6%) that passed QC were used in the WssGBLUP analysis. A complete list of proportions of additive genetic variance for fat content explained by all genomic windows is provided in Table S1. Of them, a total of 137 genomic sliding windows explaining at least 2% (arbitrary value) of the additive genetic variance for fat content are listed in Table S2. Most of the SNP sliding windows (*n* = 124; ~ 91%) were located within 62 protein-coding genes. Genomic loci affecting the additive variance for fat content were clustered in 5 chromosomes (1, 4, 5, 19, and 29) (Fig. 2).

Chromosome 19 harbored the highest number (*n* = 50) and the most significant peaks affecting fat content (up to 5.51%) (Table S2, Fig. 2). Many of the SNPs were located within the CDS of the SNP-harboring genes (*n* = 58) as well as their 3’UTR (*n* = 55). In order to understand the biological significance of the QTL associated with fat content, we annotated the SNP-harboring genes and searched their functions in the literature (described below).

**Fig. 3:**
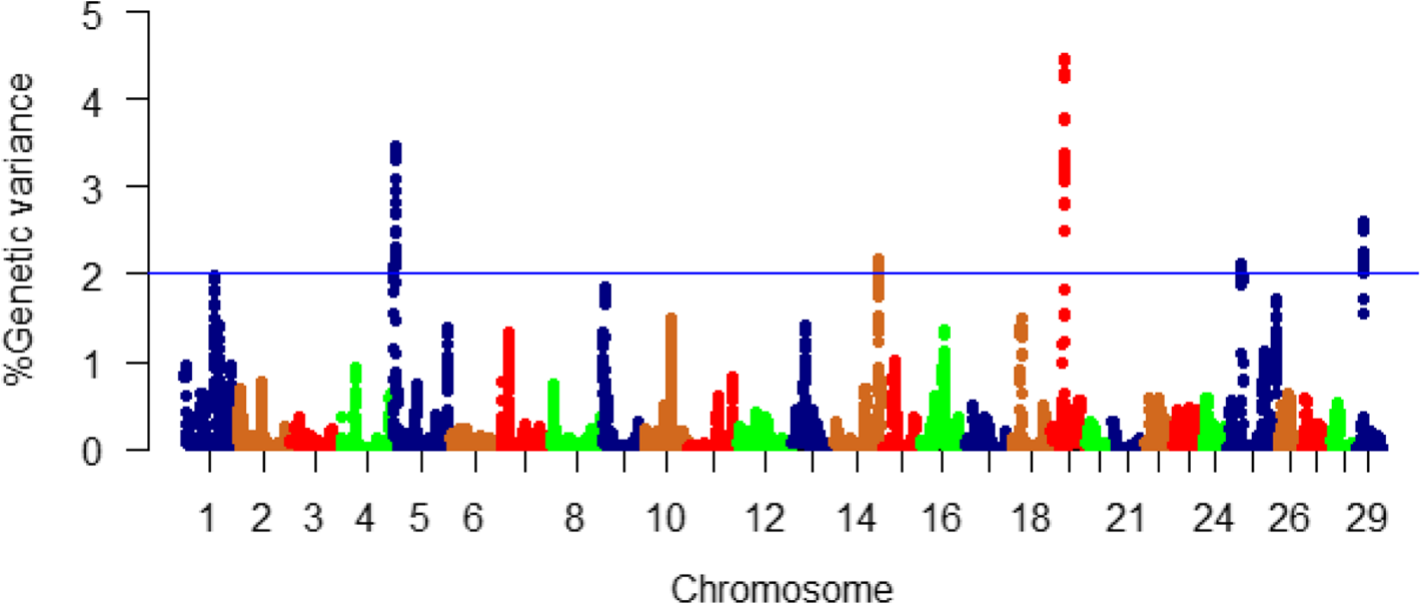
Manhattan plot showing association between 50 SNP-genomic sliding windows and muscle moisture content. Chromosome 19 showed the highest peaks with genomic loci explaining up to 4.46% of the additive genetic variance. The basal blue line represents 2% of the genetic variance explained by the sliding windows

Similarly, a complete list of the proportions of additive genetic variance for moisture content explained by all windows identified in this study is provided in Table S3. A total of 178 genomic sliding windows revealing at least 2% of the additive genetic variance for moisture content are listed in Table S4. Most of the SNP sliding windows (*n* = 165; ~ 93%) were located within 86 genes coding for proteins. Genomic loci affecting the additive variance for moisture content were clustered on 5 chromosomes (5, 14, 19, 25, and 29) (Fig. 3). Chromosome 29 harbored the highest number (*n* = 48), whereas the most significant peaks affecting moisture content (up to 4.46%) were identified on chromosome 19 (Table S4, Fig. 3). Many of the SNPs were located within CDS of the SNP-harboring genes (*n* = 68) as well as their 3’UTR (*n* = 72).

#### Common genes affecting muscle fat and moisture content

As shown above, a negative linear relationship has been established between fat and moisture content in this selectively bred rainbow trout population (YC 2010 and YC 2012), suggesting a common mechanism underlying the genetic variation in the two traits. This negative correlation was consistent with other studies in fish and mammals [23, 24, 26, 32]. In rainbow trout, the correlation between fat and moisture content depends on the physiological status of the fish. For instance, gravid fish approaching spawning and maintained on a high plane of nutrition showed reduced intramuscular fat with a concurrent increase in moisture, shear force, and protein content [26]. On the other hand, fat content was not affected during spawning, while moisture content increased [25]. This was explained by a selective mobilization of either fat or protein during sexual maturation. Depleted macromolecules were replenished by water [25, 26]. In addition to sexual maturation, season, feeding, starvation, temperature, salinity, and selection for WBW were found to affect the fat/moisture balance and impact the product quality. For instance, increased fat content due to fast growth of salmon in the summer was accompanied by a high drip loss [49]. The drip loss negatively impacted the sensory attributes and developed unpleasant odors [49]. Exposure of juvenile salmon to a high salinity stimulated lipid depletion that was partially explained by increased depot lipase activity [50]. Channel catfish fed supplemental diets deposited fat concomitant with loss of moisture. Starved channel catfish at 8.9 °C mobilized muscular fat to supply energy for metabolic process, whereas both fat and protein were mobilized at 21.1 °C; in either case, moisture content increased [51]. Selection for WBW in rainbow trout [30] and gilthead seabream [32] led to high muscular fat content associated with a decline in moisture content (in particular, the more loosely bound water).

The current WssGBLUP identified common SNPs affecting the additive genetic variance for fat and moisture content on chromosomes 19 and 29 (Tables S2 & S4). The majority of the common SNPs (*n* = 47) were located on chromosome 19. Thirty-two SNPs, out of 47, involved in lipid metabolism were identified in 16 protein-coding genes on chromosome 19 (Table 1). Briefly, cathepsin B had a single 3’UTR SNP. Cathepsin B regulates very-low-density lipoprotein (VLDL) secretion and free fatty acid uptake in response to oleic acid exposure in mice [52]. Thioredoxin-related transmembrane protein 1-like (TMX1) had three SNPs. Loss of TMX increases lipid peroxidation in TMX(−/−) mice, which, in turn, enhances oxidative stress [53]. Guanine nucleotide-binding protein GI/GS/GO gamma-2 subunit (GNG2) had a single 3’UTR SNP. GNG2 expression is positively correlated with adipocyte size [54]. SNPs in genes encoding beta-taxilin and Alpha-L-fucosidase 2 (FUCA2) were covering windows explaining the highest proportion of the additive genetic variation for fat and moisture content. Adipose tissue of obesity susceptible and resistant rats differentially expressed beta-taxilin under a high-fat diet [55]. FUCA2 is a glycolipid processing enzyme [56]. Two SNPs in F-box only protein 30 (FBXO30) and the microtubule-binding protein ensconsin were ranked next to beta-taxilin and FUCA2. An SNP in FBXO30 was located in a genomic region, explaining 4.95% of the additive genetic variance for polyunsaturated fatty acids in cattle [57]. Knockdown of microtubule-binding or -associated proteins led to changes in fat accumulation during adipogenesis [58]. Dihydropyrimidinase-related protein 5-like (CRMP5) had a single synonymous SNP. CRMP5 has GO terms belong to lipid metabolic processes [59]. Five SNPs were identified in a gene encoding trifunctional enzyme subunit alpha, mitochondrial (HADHA). This gene is involved in fatty acid beta-oxidation [59]. mRNA decay activator protein ZFP36L1 had three SNPs in the 3’UTR. Knockdown of mammalian ZFP36L1 led to the downregulation of ERK activation and inhibition of adipogenesis [60]. A single 3’UTR SNP was identified in ELM2 and SANT domain-containing protein 1 (ELMSAN1). Epigenome-wide association analysis showed DNA methylation changes in ELMSAN1 were associated with body mass index (a key measure of adiposity) [61]. Prostaglandin reductase 2 (PTGR2) had two nonsynonymous SNPs. This enzyme catalyzes reduction of the conjugated α,β-unsaturated double bond of 15-keto-PGE2 in an NADPH-dependent manner, which is a critical step in inhibition of PPARγ-mediated adipocyte differentiation [62]. Spectrin beta chain, erythrocytic (SPTB) gene had two SNPs. The SPTB interacts with phospholipids in natural [63] and model membrane systems [64] and has a role in controlling the fluidity of the inner lipid leaflet of the cell membrane (reviewed in [65]). 
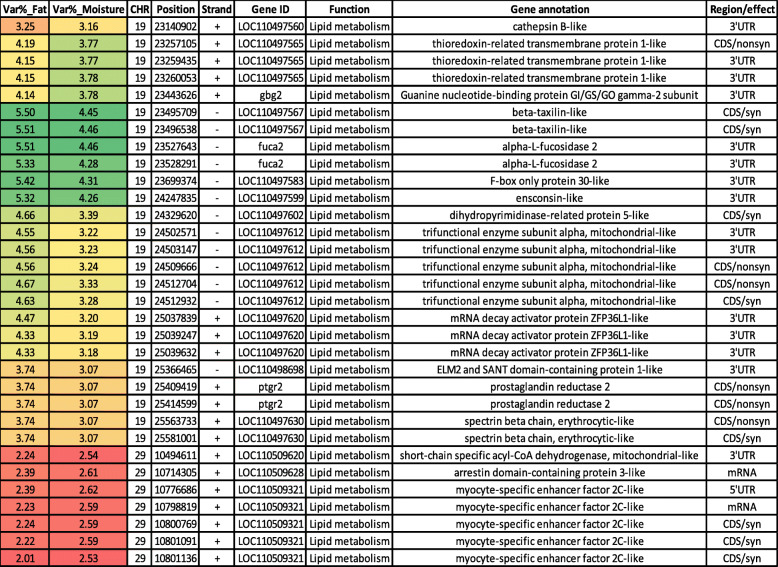

**Table 1:**
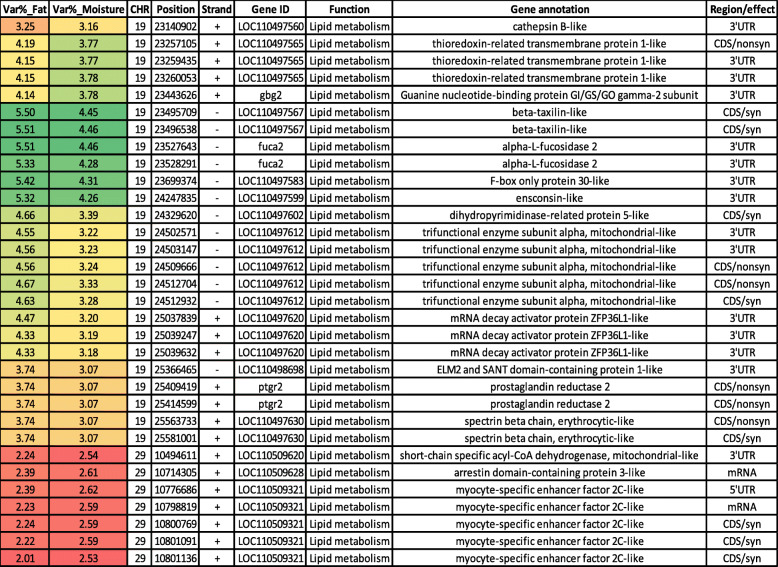
SNP markers in genomic sliding windows explaining at least 2% of the genetic variance for fat and moisture content and involved in lipid metabolism. A color gradient on the left indicates differences in additive genetic variance explained by windows containing the representative SNP marker (green is the highest and red is the lowest). SNPs are sorted according to their chromosome positions

Chromosome 29 had 14 SNPs in genomic windows explaining at least 2% of the additive genetic variance for intramuscular fat and moisture content (Tables S2 & S4). Of them, seven SNPs were involved in lipid metabolism (Table 1). A gene encoding short-chain specific acyl-CoA dehydrogenase, mitochondrial (ACADS) had a single SNP. This enzyme has a role in fatty acid beta-oxidation [66]. An intronic SNP was identified in a gene coding for arrestin domain-containing protein. The latter has GO terms belonging to fat pad and skin development and regulates the body mass [67]. Myocyte enhancer factor 2c (MEF2C) had the highest number of SNPs (*n* = 5) on chromosome 29. MEF2C is a transcription factor involved in skeletal muscle differentiation; however, it has been reported as a constituent of a mechanism that programs gene expression involved in development of brown adipocytes [68]. MEF2A and MEF2D isoforms exhibited in vivo differential expression in mammalian striated muscle and white adipose tissue of insulin-deficient diabetic mice [69]. To our knowledge, the role of MEF2C in white adipose tissue remains uncertain.

In addition, twelve SNPs in genes involved in transmembrane transport and cytoskeleton remodeling were identified in common QTL affecting additive variance for fat and moisture content (Table 2). The majority of these SNPs were identified on chromosome 19 (*n* = 11). Three synonymous SNPs were identified in a gene encoding intersectin-2 (ITSN2). This protein is necessary for the clathrin-mediated endocytosis and actin cytoskeleton remodeling [70]. Six SNPs were identified in 3 genes involved in vesicle-mediated transport (i.e., exocytosis); dnaJ homolog subfamily C member 5B, visinin-like protein 1, and syntaxin-binding protein 5. The actin cytoskeleton remodeling controls each step of exocytosis [71]. Three SNPs were identified in microtubule-associated protein RP/EB family member 3 (MAPRE3) and centrin-3. MAPRE3 and centrin-3 control the dynamics of the microtubule cytoskeleton [59, 72]. 
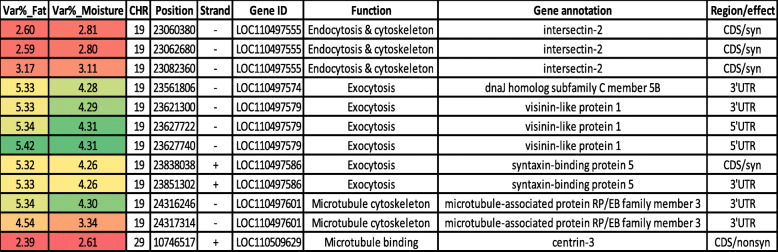

**Table 2:**
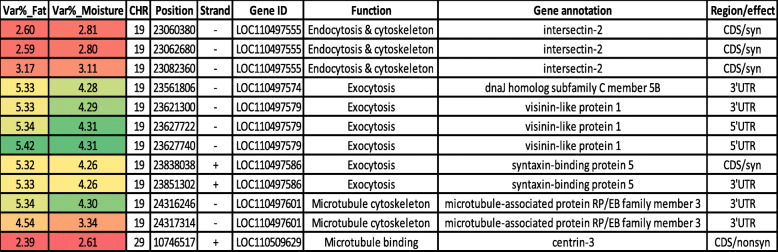
SNP markers in genomic sliding windows explaining at least 2% of the genetic variance for fat and moisture content and involvement in transmembrane transport and cytoskeleton regulation. A color gradient on the left indicates differences in additive genetic variance explained by windows containing the representative SNP marker (green is the highest and red is the lowest). SNPs are sorted according to their chromosome positions

Overall, the analysis revealed that most of the loci in association with both fat and moisture content are involved in lipid metabolic process. Therefore, our results suggest that variation in moisture content is likely to be driven by changes in fat content in an antagonistic fashion. This notion was previously suggested as depletion of macromolecules under catalytic muscle conditions is likely forming voids for water accumulation [28].

#### Unique genes affecting the additive genetic variance for moisture

The actin cytoskeleton interacts with the cell membrane to control water transport [73]. Expression of genes involved in the cytoskeletal organization has previously shown a positive correlation with the drip loss [74]. In the current study, thirty-five variants in genes (*n* = 14) involved in cytoskeleton remodeling were identified, affecting the additive variance for moisture content in rainbow trout (Table 3). Briefly, bone morphogenetic protein receptor type-2 (BMPR2) had a single synonymous SNP. BMPR2 is known to interact with the cytoskeleton, and BMPR2 mutant mice exhibited cytoskeletal defects [75]. A gene encoding muscle associated receptor tyrosine kinase (MUSK) had a single SNP. Activation of MUSK in myotubes regulates the reorganization of the actin cytoskeleton [76]. Two SNPs were identified in THAP domain containing 1 (THAP1), which has a role in regulation of the mitotic cell cycle [77]. The gene encoding asparaginyl-tRNA synthetase (NARS) had 3 SNPs in windows explaining the highest additive variance (up to 3.46%; Table 3). Mutations in NARS leads to cell cycle arrest in the S phase [78]. The actin cytoskeleton undergoes dramatic changes during the cell cycle [79]. Ten SNPs were identified in three genes coding for cyclin-I (CCNI), cyclin-G1 (CCNG1), and cyclin-G2 (CCNG2). Cyclins function as regulators of the cell cycle and actin cytoskeleton dynamics (reviewed in [80]). The serine/threonine-protein, phosphatase 2A (PP2A), had a 3’UTR SNP. This phosphatase is associated with microtubule stabilization, where it binds and dephosphorylates the microtubule-associated proteins [81]. Annexin A6 (ANXA6) had two synonymous SNPs. ANXA6 contributes to membrane and cytoskeleton organization in a Ca^2+^-dependent manner [82]. Tubulin beta-4B chain (TUBB4B) had four synonymous SNPs within 1Kb of chromosome 25. TUBB4B is a critical component of microtubules [59]. Five SNPs, clustered in ~2Kb, were identified in a gene coding for mid1-interacting protein 1 (MID1IP1). This protein enhances fatty acid biosynthesis [83] and stabilizes microtubule organization [59]. Two SNPs were identified in a gene encoding tubulin-specific chaperone A (TBCE). TBCE is a tubulin-folding protein required for proper microtubule cytoskeleton organization [84]. Additionally, mutations in TBCE drive muscle atrophy [84]. Proteinase-activated receptor 1 (PAR1) and PAR2 had four SNPs. PAR-mediated, RhoA activation is vital for cytoskeletal reorganization [85]. 
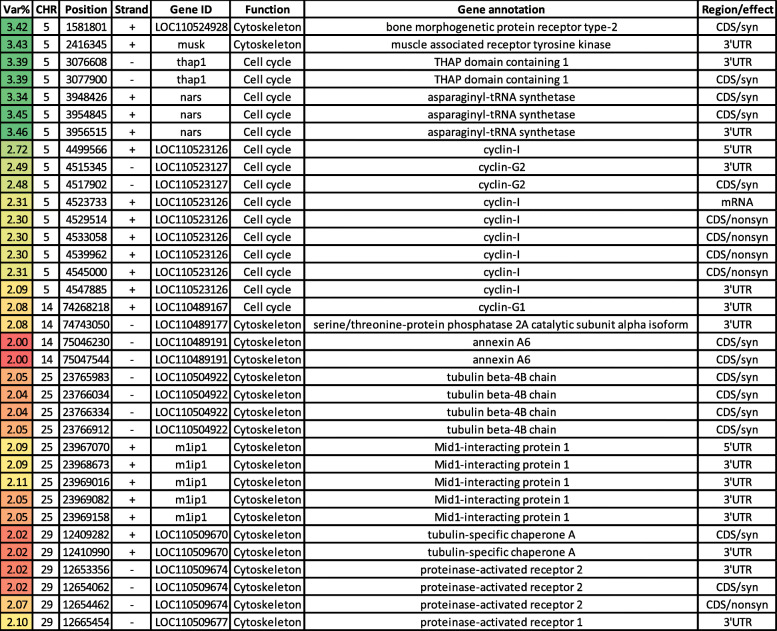

**Table 3:**
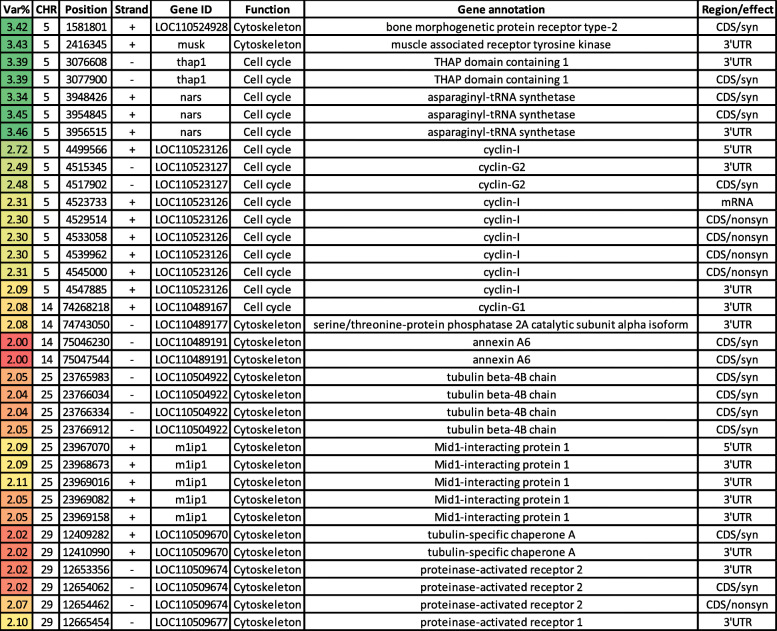
SNP markers in genomic sliding windows explaining at least 2% of the genetic variance for moisture content and involved in cell cycle and cytoskeleton regulation. A color gradient on the left indicates differences in additive genetic variance explained by windows containing the representative SNP marker (green is the highest and red is the lowest). SNPs are sorted according to their chromosome positions

A strong positive correlation between moisture and protein content has been established in different species. A simultaneous decline in protein and moisture content was previously reported in mammals [24]. Moisture content in rainbow trout exhibited a bidirectional relationship with protein content depending on physiological/metabolic status. For example, a negative correlation between moisture and protein content were previously reported under muscle catabolic conditions associated with full sexual maturation (R^2^ = 0.994, *p* < 0.01) [25]; whereas, a positive correlation was reported in female trout, on a high plane of nutrition, that were approaching spawning [26]. This was explained by selective mobilization of either protein during spawning or fat before spawning; in either case, the depleted macromolecule was replaced by water. It is noteworthy that protein content variation of the current study was not statistically significant between the 4 high-ranked families versus 4 low-ranked families (data not shown). The current WssGBLUP analysis indicated that thirteen SNPs in genes involved in protein degradation were involved in the additive genetic variance of moisture content (Table 4). Briefly, E3 ubiquitin-protein ligase RNF170 is an E3 ubiquitin-protein ligase that plays an essential role in the ubiquitination and degradation of inositol 1,4,5-trisphosphate receptor type 1 (ITPR1) [59, 86]. The latter controls the calcium release from the endoplasmic reticulum [87], which affects the muscle protein content in rainbow trout [39] and has a profound effect on the regulation of cytoskeleton [88]. Cystatin-1, which possesses a peptidase inhibitor activity, had a single 5’UTR SNP. Thioredoxin-like 1 (TXNL1) had two synonymous SNPs. The knockdown of TXNL1 moderately stabilizes the ubiquitin-protein conjugates suggesting a connection between protein reduction and proteolysis [89]. Pre-mRNA-processing factor 19 (PRPF19) and ubiquitin-conjugating enzyme E2 D2 (UBE2D2) had four SNPs. These ligases catalyze polyubiquitin chain assembly and play a role in proteasomal protein degradation [90, 91]. Nuclear factor NF-kappa-B p105 subunit (NFKB1) had two 3’UTR SNPs. NFKB1 is involved in the negative regulation of cellular protein metabolic process [92] and apoptotic process [93]. 
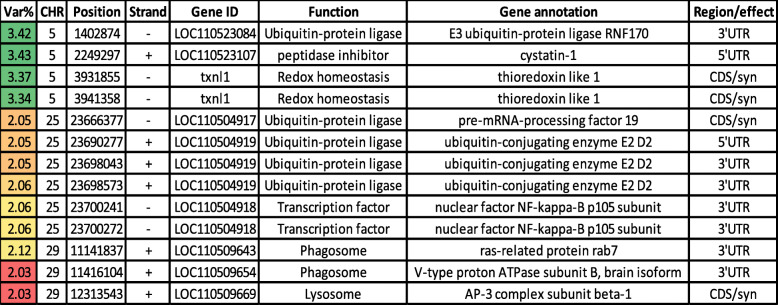

**Table 4:**
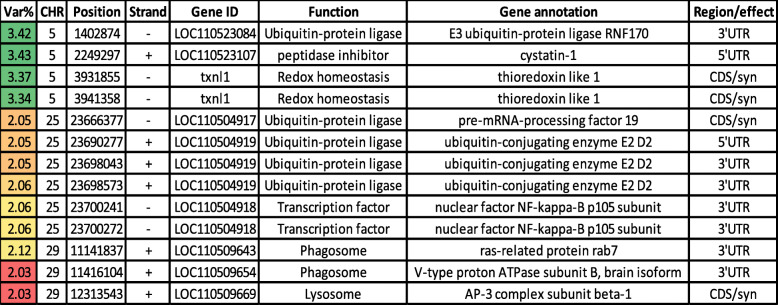
SNP markers in genomic sliding windows explaining at least 2% of the genetic variance for moisture content and involved in proteolytic activities. A color gradient on the left indicates differences in additive genetic variance explained by windows containing the representative SNP marker (green is the highest and red is the lowest). SNPs are sorted according to their chromosome positions

In addition to the ubiquitin-protein ligases, SNPs in three genes involved in lysosomal/phagosomal pathways were identified. Ras-related protein rab7 (RAB7A) harbored a 3’UTR SNP. RAB7A is a major regulator of endo-lysosomal maturation/ trafficking and protein targeting to lysosome inducing autophagosome formation [94]. Thus, RAB7A positively regulates the protein catabolic process [95]. V-type proton ATPase subunit B (ATP6V1B2) had a 3’UTR SNP. V-ATPase is responsible for acidifying the intracellular compartments, including lysosomes [96]. The gene encoding the β chain of the adaptor protein-3 (AP-3) complex had a single synonymous SNP. Deletion in AP3B1 perturbs assembly of AP-3 complex and, in turn, trafficking of transmembrane lysosomal proteins [97].

In this study, most of the common genomic loci affecting the highest proportion of the additive variance were involved in lipid metabolism, suggesting a common mechanism underlying intramuscular fat and moisture content and, partially, explaining the strong negative correlation between the fat and moisture content in this selectively bred rainbow trout population. Unique loci affecting moisture content were primarily involved in cytoskeleton regulations and protein turnover. Inhibition of protease activity, such as calpains, reduced degradation of proteins responsible for cell membrane-cytoskeleton attachments and postmortem drip channel formation in muscle [98]. The presence of calcium enhances proteolysis, by μ-Calpain, of myofibrillar and other cytoskeletal proteins during postmortem storage [45, 99]. Further investigation is warranted to determine QTL that could be prioritized in breeding programs to achieve optimal moisture content with low enzymatic activity and drip loss, and optimal fat content that can meet consumer preferences.

Overall, the WssGBLUP analysis has enriched the current understanding of the genetic architecture of the fat and moisture content in rainbow trout. Common SNP windows explained a high proportion of the additive genetic variance associated with both fat and moisture content, suggesting common regulatory mechanisms. Knowledge of the heritability of fat and moisture content and their correlations with other traits is needed for establishment of sustained multi-trait selection programs. The aquaculture industry is interested in implementing genomic selection in the breeding programs; however, applying high-density SNP chips is cost-prohibitive for small-sized hatcheries and companies. SNPs with a large-effect on genetic variances of fat and moisture content, identified in this study, could be prioritized to reduce SNP panel density needed to evaluate the predictive abilities for both traits. In another study, we found that prioritizing SNPs based on the proportion of variance explained for muscle yield and firmness allowed to reduce the SNP panel density down to ~ 800 SNPs. Reduced SNP panels outperformed the traditional PBLUP model in predicting the future fish performance, and maintained predictive abilities comparable to the 50 K SNP panel (data will be published elsewhere).

### Single marker GWA analyses

**Fig. 4:**
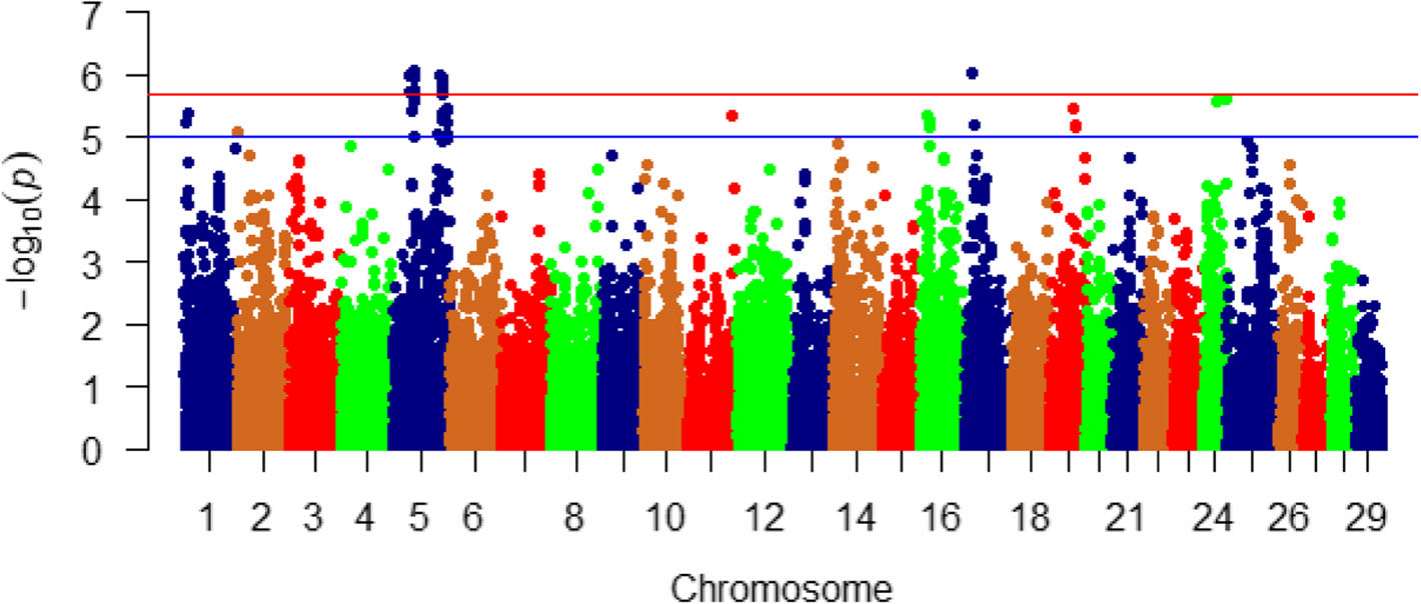
Manhattan plot showing single SNP markers significantly associated with variation in muscle fat content using family-based association analysis. Many of the significant SNPs are located on chromosome 5. Blue and red horizontal lines represent suggestive (1e-05) and significant (1.69e-06) threshold *p*-values, respectively

**Fig. 5:**
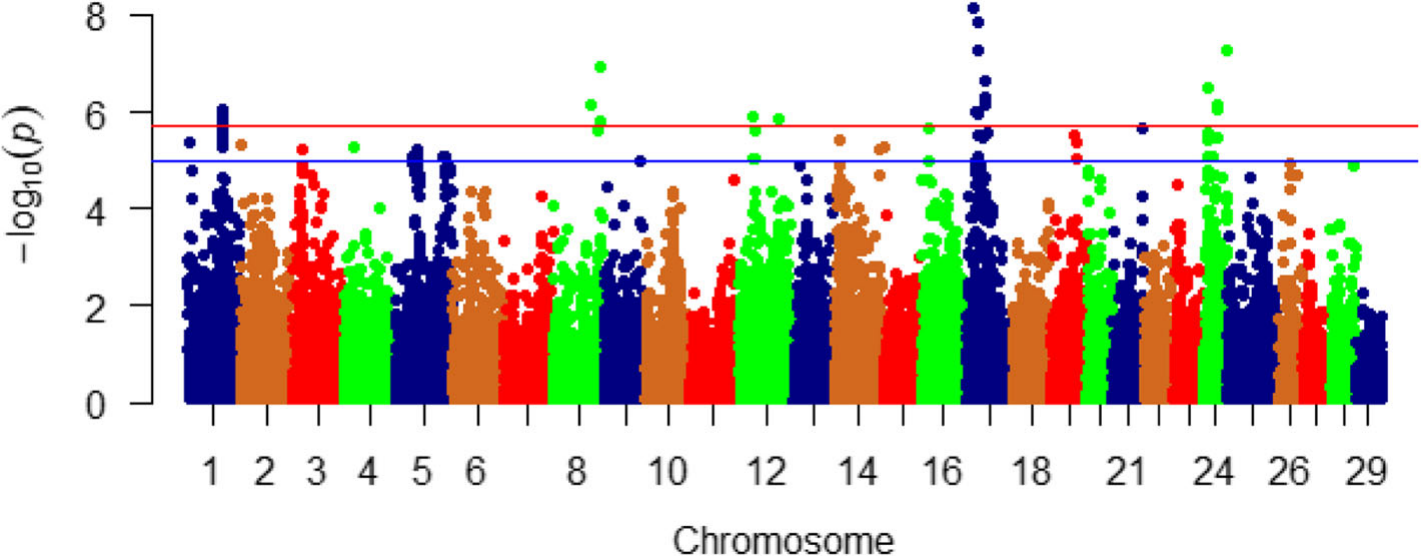
Single SNP markers showing significant associations with variation in moisture content using family-based association analysis. Most of the significant SNPs are located on chromosome 17. Blue and red horizontal lines represent suggestive (1e-05) and significance (1.69e-06) threshold *p*-values, respectively

To identify single SNP markers associated with variation in fat and moisture content, we analyzed SNPs that passed QC filtration (*n* = 29,451) using a generalized score test; this test incorporates multiple covariates in the analysis and accounts for family structure using a kinship matrix [100]. In this study, 8 and 24 significant SNPs, surpassing the genome-wide significance level, had a potential impact on the fat and moisture content (Bonferroni-corrected *p* < 1.69E-06; Figs. 4 and 5 and Tables S5 & S6), respectively. Whereas, 29 and 46 SNPs surpassing the suggestive significance level (Bonferroni-corrected p < 1E-05; Figs. 4 and 5 and Tables S5 & S6) were detected in association with fat and moisture content, respectively. Suggestive significant SNPs were not considered for the downstream analysis.

SNPs associated with the fat content were mainly located on chromosome 5 (*n* = 7), and SNP-harboring genes have roles in lipid metabolism (Table 5). The list includes 78 kDa glucose-regulated protein (GRP78), spindle and kinetochore associated complex subunit 1 (SKA1), apelin receptor B (APLNR-B), desmoplakin, podocan, and calcium-binding mitochondrial carrier protein SCaMC-1 (SLC25A24). Briefly, two missense mutations were identified in genes coding for GRP78 and SKA1. GRP78 is essential for adipocyte differentiation and a balanced secretion of adipokines. Deletion of GRP78 causes lipoatrophy in mice observed as a dramatic reduction in gonadal and subcutaneous adipose tissue [101]. SKA1 was downregulated in adipose tissues between samples from obese and healthy control children and has been suggested as a candidate biomarker for childhood obesity [102]. Three synonymous mutations were identified in genes encoding APLNR, desmoplakin, and podocan. APLNR knockout mice demonstrated excess fatty acid accumulation in skeletal muscle [103]. Abnormalities in desmoplakin have been associated with changes in lipid metabolism [104]. Podocan belongs to the small leucine-rich proteoglycans (SLRPs) that bind to low-density lipoprotein receptor-related protein (LRP-1) [105]. A 3’UTR SNP was identified in a gene coding for SLC25A24. Mice fed a high-fat diet exhibited increased expression level of SLC25A24; whereas, adipocyte differentiation was suppressed in Slc25a24- knockout [106]. 
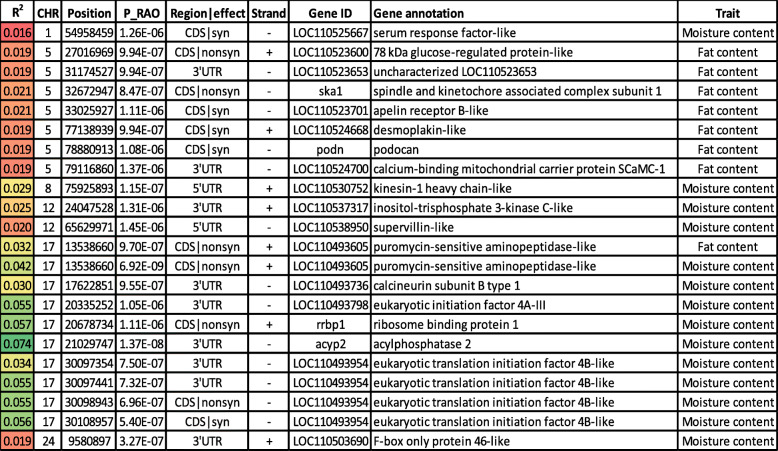

**Table 5:**
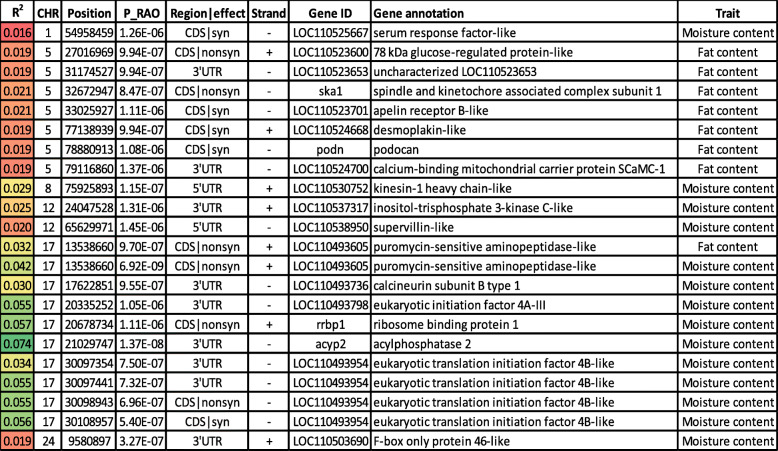
SNP markers significantly associated with variability in fat and moisture content using family-based association analysis. A color gradient on the left shows the phenotypic variation explained by each SNP marker (green is the highest and red is the lowest). SNPs associated with the phenotypes are sorted according to their chromosome positions

Puromycin-sensitive aminopeptidase (NPEPPS), on chromosome 17, had a nonsynonymous SNP explaining the highest variability in fat content (R^2^ = 3.2%) (Table 5). NPEPPS impacts different physiological processes, including protein turnover and cell cycle regulation. NPEPPS was upregulated in mitten crabs fed with a linseed oil rich in linoleic acid [107]. However, the effect NPEPPS on lipid metabolism in fish needs further investigations.

SNPs associated with moisture content (*n* = 24) were associated with protein turnover, calcium metabolism, and cytoskeleton integrity (Table S6). Most of these SNPs (*n* = 11; ~ 46%) were located on chromosome 17. An SNP in a gene coding for acylphosphatase 2 ranked at the top of the list (R^2^ = 7.4%) (Table 5); however, its physiological role is not clear. Eight SNPs associated with moisture content were identified in five genes engaged in protein metabolism (Table 5). These genes are NPEPPS, eukaryotic initiation factor 4A-III (EIF4A3), eukaryotic translation initiation factor 4B (EIF4B), ribosome binding protein 1 (RRPB1), and F-box only protein 46 (FBXO46). Briefly, the aminopeptidase, NPEPPS, was associated with variation in fat and moisture content, suggesting a correlation between moisture and fat content. Five SNPs were identified in two genes encoding EIF4A3 and EIF4B, suggesting a role for the protein translation machinery in determining variation in moisture content. RRPB1 is an ER integral membrane protein implicated in polysome assembly and, therefore, protein synthesis [108]. RRPB1 has been suggested as essential in regulation of UPR signaling molecules and autophagy [109]. Finally, the F-box family SCF-E3 ubiquitin ligase, FBXO46, had a single 3’UTR SNP.

A previous report showed that expression of genes involved in the actin cytoskeleton and cytoskeletal organization is positively correlated with drip loss in pig [74]. In this study, a total of 10 SNPs were associated with moisture content were identified in seven genes engaged in cytoskeleton regulation (Table 5). These genes are encoding serum response factor (SRF), kinesin-1 heavy chain (KIF5B), inositol-trisphosphate 3-kinase C (IP3KC), supervillain (SVIL), calcineurin subunit B type 1 (PPP3R1), eIF4A and EIF4B. Briefly, SRF is a master regulator of the actin cytoskeleton [110]. Mutations in KIF5A caused cytoskeletal defects in humans [111]. Two, 3’UTR SNPs were identified in IP3KC and PPP3R1. IP3K and calcium/calcineurin signaling play critical roles in maintaining Ca^2+^ homeostasis that has a profound effect on the cytoskeleton [88, 112, 113]. Supervillin (SVIL) is one of the first components of the costameric membrane skeleton to assemble during muscle formation. It establishes a high-affinity connection between the membranes and actin cytoskeleton [114]. Translation initiation factors, including eIF4A and EIF4B, associate with the actin cytoskeleton, which affects protein synthesis [115].

In our previous work, we profiled transcriptome expression of fish families (YC 2010) showing contrasting phenotypes in fat content, which revealed only 17 differentially expressed transcripts associated with fat content [31]. About 90% of the genetic variation among individuals comes from SNPs [116], and therefore, identifying SNP markers associated with complex traits is most suitable for genetic evaluation in selection programs. Few previous GWA studies identified a small number of SNPs responsible for the additive variance for fat content in Atlantic salmon and Common Carp [11, 34, 38]. The current GWA analysis identified a total of 137 SNPs in windows explaining at least 2% of the additive genetic variance for fat content, suggesting a better characterization of the genetic basis underlying variation in fat content. The discrepancies among the different GWA studies might be due to; 1) usage of different algorithms in the GWA studies, 2) variation in population size, 3) substantial difference in the capacity of the SNP arrays, 4) polygenic nature of intramuscular fat content, 5) different thresholds in each study including sliding window size [35].

Compared to our WssGBLUP analysis, the single marker GWA analysis revealed a smaller number of SNP markers associated with variation in intramuscular fat and moisture content. Besides, these two GWA approaches revealed different significant peaks associated with traits of interest. Aguilar et al. [117] showed that the highest peak based on the *p*-value was not the same based on the proportion of variance explained, and this is because the latter depends on allele frequency, i.e., high effect but low frequency decreases the variance explained. This result is consistent with other studied traits, such as fillet firmness, protein content [39], and bodyweight gain [40] in rainbow trout. The potential factors associated with observed heterogeneity between the two approaches are different algorithms, thresholds, and windows size used in each approach. For instance, WssGBLUP uses a flexible default HWE threshold as it assumes selection may have caused a departure from equilibrium, and therefore only extreme outliers would be excluded in order to keep the information content of haplotypes. The WssGBLUB was more effective than the single marker GWA in examining the genetic architecture of studied traits and identifying common QTL between traits. This method has proven to be optimal for breeding populations given the data structure: phenotyped individuals may not have genotypes, and there is a long history of pedigree recording [117]. Common QTL identified in this study may explain the high negative correlation between fat and moisture content. The recombinational progression of QTL and nearby markers determines the information content of haplotypes [118]. However, SNP-harboring genes identified by the two approaches had similar biological functions and were involved in lipid metabolism, protein turnover, and cytoskeletal remodeling. Routine use of single-SNP and multi-makers for GWA analysis was previously recommended to take advantage of the complete information content of the genotypes [118].

Taken together, controlling muscular fat content can help the aquaculture industry to produce a final product of expected quality, including moisture content, drip loss, firmness, and shelf-life. Dietary lipids are used to increase fillet fat to improve fillet sensory characteristics [119]. However, this can also elevate the feeding costs, increase visceral fat, and accelerate lipid oxidation, which increases fillet degradation. Alternatively, genetic/genomic selection can be used to control fillet fat content. The findings of the current study can help breeders where GEBV for muscular fat and moisture content can be added to multi-trait selection indices that reflect the various needs of producers and consumers [120].

## Conclusions

The current GWA analyses identified novel genomic regions associated with additive genetic variance for fat and moisture content in rainbow trout. SNP-harboring genes encode proteins with a role in lipid metabolism, actin cytoskeleton remodeling, and protein synthesis/degradation. This work reveals significant QTL associated with fat content, which appears to be a polygenic trait. The top common windows affecting additive genetic variance for fat and moisture content are mainly on chromosome 19. These findings provide a genetic basis for description of the molecular mechanisms underlying fat and moisture content in teleost fish. Variation in moisture content is likely to be driven by changes in fat content in an antagonistic fashion. This work provides putative markers that could be prioritized when estimating genomic breeding values for fat and moisture content. GEBV for muscular fat and moisture content can be included in multi-trait selection indices that meet the various producers’ and consumers’ demands [121].

## Methods

### Fish population, tissue sampling, and phenotypic traits

The fish population used in the current GWA analyses was previously described in [122]. Briefly, a five-generation selective breeding program was established at NCCCWA in 2002 by intercrossing seven domesticated strains of rainbow trout; this fish population was selected for improved growth performance [30]. Phenotypic data for muscle fat and moisture content were obtained from 789 fish representing 197 full-sib families produced from two consecutive generations (YC 2010 and 2012). Single-sire×single-dam matings occurred over 6 weeks to produce full-sib families. Individuals from each family were reared together in a 200-L tank in order to keep the pedigree information. Tagging fish at ~ 5-months post-hatch allowed to rear different fish families together in 800-L communal tanks until ~ 13 months post-hatch. Fish were starved for 5 days before harvest to facilitate viscera removal.

Over five weeks, a single fish/family was collected and randomly assigned to a harvest group as described in [40]. Fish from YC 2010 were younger than those from YC 2012. Fish were euthanized (300 mg of MS-222 per liter), harvested, and eviscerated. Afterwards, the carcasses were hand-made into skinless fillets.

Proximate analyses, including crude lipid and moisture content (water content), were previously described [123]. Crude lipid content was determined using Soxhlet extraction with petroleum ether, whereas moisture content was assessed by the loss on drying method. When muscle fat and moisture content were regressed on body weight, coefficient of determination (R^2^) values of 0.23 and 0.38 were observed, respectively. Heritability was estimated for fat and moisture content using a genomic relationship matrix (GRM) [100].

### SNP genotyping and quality control

A 50 K, transcribed gene SNP-chip was recently developed and used in identifying genomic loci responsible for additive genetic variance in fillet yield [36]. SNPs utilized to construct the SNP chip were reported in our previous study [122]. The chip included ~ 5 K nonsynonymous SNPs and ~ 21 K SNPs exhibiting potential allelic imbalances with economic traits [36, 122]. Other SNPs were added to the chip to reach a total of 50,006 SNPs with a minimum of 2 SNPs per SNP-harboring gene.

In total, 1728 rainbow trout fish were used for genotyping and quality assessment of the SNP chip. Genotyped samples were filtered by the SNPolisher software using a call rate threshold of 0.97 and Dish QC cutoff of 0.82 [36]. Genotyped fish with records for intramuscular fat and moisture content (789 fish), were used for the current GWA studies.

### Fifty-SNP window GWA analysis

Estimates of SNP effects from WssGBLUP were utilized to conduct the current GWA analyses, as described in [36]. The WssGBLUP combines phenotypes, genotypes, and pedigree information into a single evaluation. GRM was created based on VanRaden equation [124]. SNPs used to create the GRM were weighted according to the proportion of additive variance they explain. The following single-trait model was used:

$$ \mathbf{y}=\mathbf{Xb}+{\mathbf{Z}}_1\mathbf{a}+{\mathbf{Z}}_2\mathbf{w}+\mathbf{e} $$

where **y** is the vector of phenotypes (fat or moisture content), **b**, **a** and **w** are vectors of fixed and random effects, and **e** is the vector of residual effects. Random and fixed effects were determined according to [35, 125]. **X**, **Z**_1_, and **Z**_2_ are incidence matrices for fixed and random effects in vectors **b**, **a**, and **w**, respectively. Whereas the family and residual random effects were considered uncorrelated, the animal effect was correlated. The covariance structure for the animal effect was given by $\mathbf{H}{\upsigma}_{\mathrm{a}}^2$, where **H** is a matrix that combines pedigree- and genomic-based relationships [126] and ${\upsigma}_{\mathrm{a}}^2$ is the additive variance.

The variance components were estimated using AIREMLF90 [127]. The inbreeding coefficient was calculated by INBUPGF90 [128], as described in [36]. Genomic data were edited using PREGSF90 [127], and samples or SNPs were kept according to the following parameters: minor allele frequency (MAF) > 0.05, a default value of Hardy-Weinberg equilibrium (HWE) < 0.15, and call rate > 0.90. In total, 35,322 SNPs (70.6%) passed the QC and were used for the WssGBLUP analyses.

The filtered SNPs (~ 35 K) were subjected to a two-iteration WssGBLUP analysis. All SNPs were assigned weight = 1.0 in the first iteration. SNP effects ($\hat{u}$) were determined using POSTGSf90 (part of GBLUPf90 software family) according to:

$$ \hat{u}= qD{Z}^{\prime }{\left( ZDZ\prime q\right)}^{-1}\ \hat{a} $$

Where q is a weight factor based on SNPs frequency, D is a weight matrix of SNPs, Z is a matrix of gene content adjusted for allele frequencies, and â is Genomic Breading Values of genotyped animals [129].

Using $\hat{u}$ and the allele frequency (*p*), SNP weights were determined as ${\hat{u}}^22p\left(1-p\right)$ in the second iteration. SNP effects and updated weights were computed by POSTGSF90 [128] using genomic sliding windows of 50 contiguous SNPs. The proportion of additive variance explained by an i-th region was computed according to [129]:

$$ \frac{\mathit{\operatorname{var}}\left({a}_i\right)}{\sigma_a^2}\ast 100\%=\frac{\mathit{\operatorname{var}}\left({\sum}_{j-1}^{50}{z}_j{\hat{u}}_j\right)}{\sigma_a^2}\ast 100\% $$

Where i is the i-th region that consists of 50 adjacent SNPs, a_i_ is the genetic value of the contiguous 50 SNP region, σ^2^_a_ is the total genetic variance, j is the j-th SNP within the 50 SNP region, Z_j_ is a vector of the gene content of an SNP for all fish, and û_j_ is the effect of an SNP within the contiguous 50 SNP region.

The qqman package [130] in R was used to generate Manhattan plots showing the percentage of genetic variance explained.

### Single marker GWA analysis

PLINK [131] was used to filter the genomic data before performing a single marker association analysis. The filtering criteria included MAF > 0.05 and a default value of HWE < 0.001. PLINK was used to retrieve R-squared values (R^2^) of association between the quantitative traits and genotypes. R^2^ is the proportion of variance in the phenotype explained by the genetic factors in a linear regression model. ONETOOL [100] was used to perform a family-based association analysis where it allows for incorporating multiple covariates and accounts for family structure. Covariates were incorporated in the linear model to account for fixed effects (harvest group and hatch-year) and population structure. Bonferroni corrected *p*-values were calculated as (α/total number of variants), where α = 0.05 was used for a genome-wide significance level, and α = 0.3 was used for the suggestive significance level. Manhattan plots showing single markers associated with variation in intramuscular fat and moisture content were generated using qqman package [130].

### Gene annotation

The genome annotation file was used to annotate the SNP-harboring genes by Bedtools [132]. SNPs were categorized as genic or intergenic depending on their physical location relative to the body of the gene. Genic SNPs exist in coding DNA sequence (CDS), introns, or untranslated regions (5’UTR and 3’UTR). Intergenic SNPs are defined as SNPs located in the region between genes.

## Supplementary information

**Additional file 1: Table S1**. Genetic variance for fat content explained by all SNP markers in genomic sliding windows. **Table S2**. SNP markers in genomic sliding windows explaining at least 2% of the genetic variance for fat content. **Table S3**. Genetic variance for moisture content explained by all SNP markers in genomic sliding windows. **Table S4**. SNP markers in genomic sliding windows explaining at least 2% of the genetic variance for moisture content. **Table S5**. SNP markers significantly associated with variability in fat content (highlighted in yellow) using family-based association analysis. **Table S6**. SNP markers significantly associated with variability in moisture content (highlighted in yellow) using family-based association analysis.

## Data Availability

All datasets generated for this study are included in the manuscript and/or the Additional Files. The genotypes (ped and .map files) are available in our previous publication [39].

## Abbreviations

BCWD: Bacterial Cold Water Disease
GO: Gene ontology
GWA: Genome-wide association
HWE: Hardy–Weinberg equilibrium
MAF: Minor allele frequency
NCCCWA: USDA National Center of Cool and Cold Water Aquaculture
QC: Quality control
QTL: Quantitative trait loci
SNP: Single nucleotide polymorphism
UTR: Untranslated region
WssGBLUP: Weighted single-step GBLUP
YC: Year class
